# Supplementary material for: DMRforPairs: identifying Differentially Methylated Regions between unique samples using array based methylation profiles
Source: BMC Bioinformatics. 2014 May 15;15:141. doi: 10.1186/1471-2105-15-141 (PMC4046028; doi:10.1186/1471-2105-15-141)
Supplement: Additional file 2 — DMRforPairs output for the comparison of A431-MCF7 and NA17018-NA17105. Please start from the HTML files in each folder. Available via the BMC Bioinformatics website. [file 1471-2105-15-141-S2.zip › 1394847754114233_MOESM2_ESM/A431_MCF7/figures/34151.pdf]

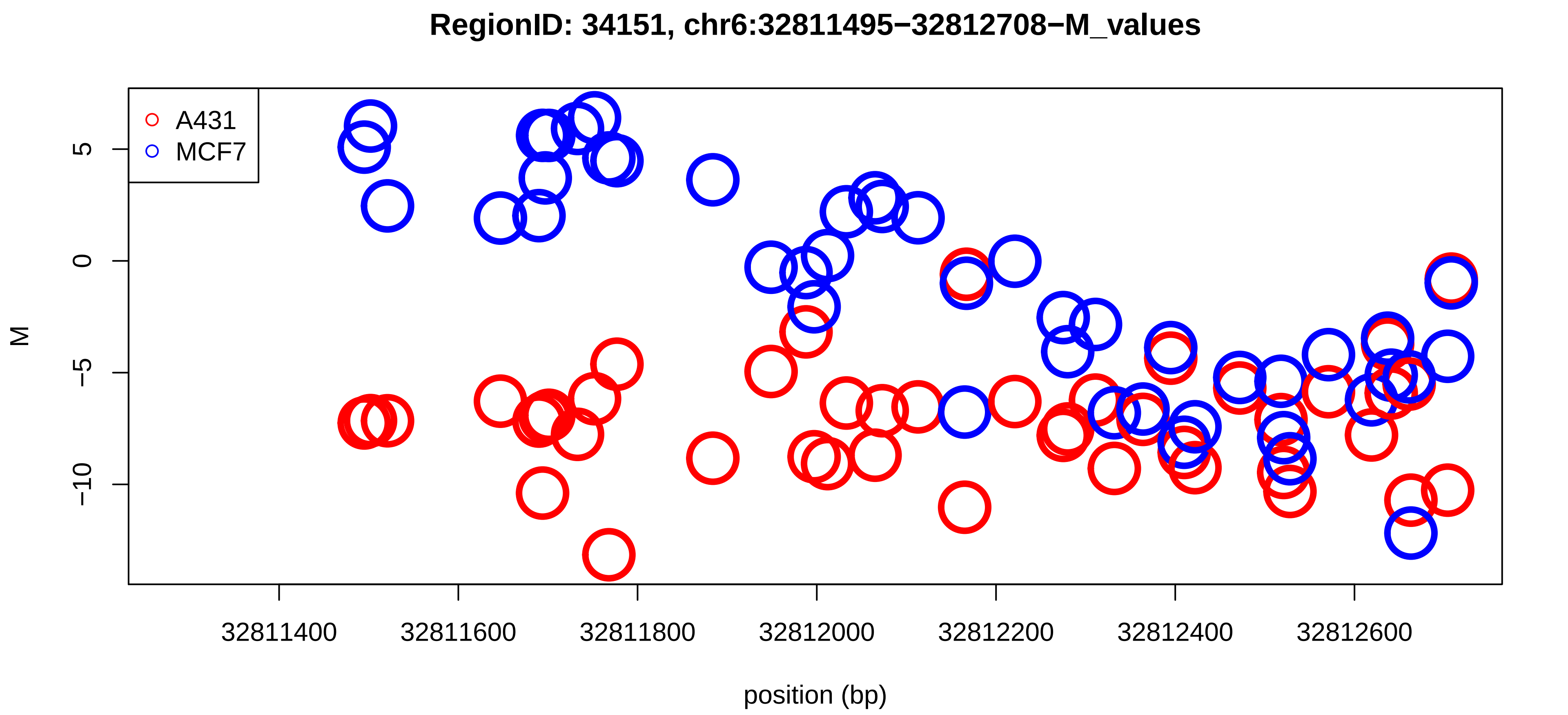

RegionID: 34151, chr6:32811495−32812708−M_values
A431
MCF7
M
5
0
−5
−10
32811400
32811600
32811800
32812000
32812200
32812400
32812600
position (bp)

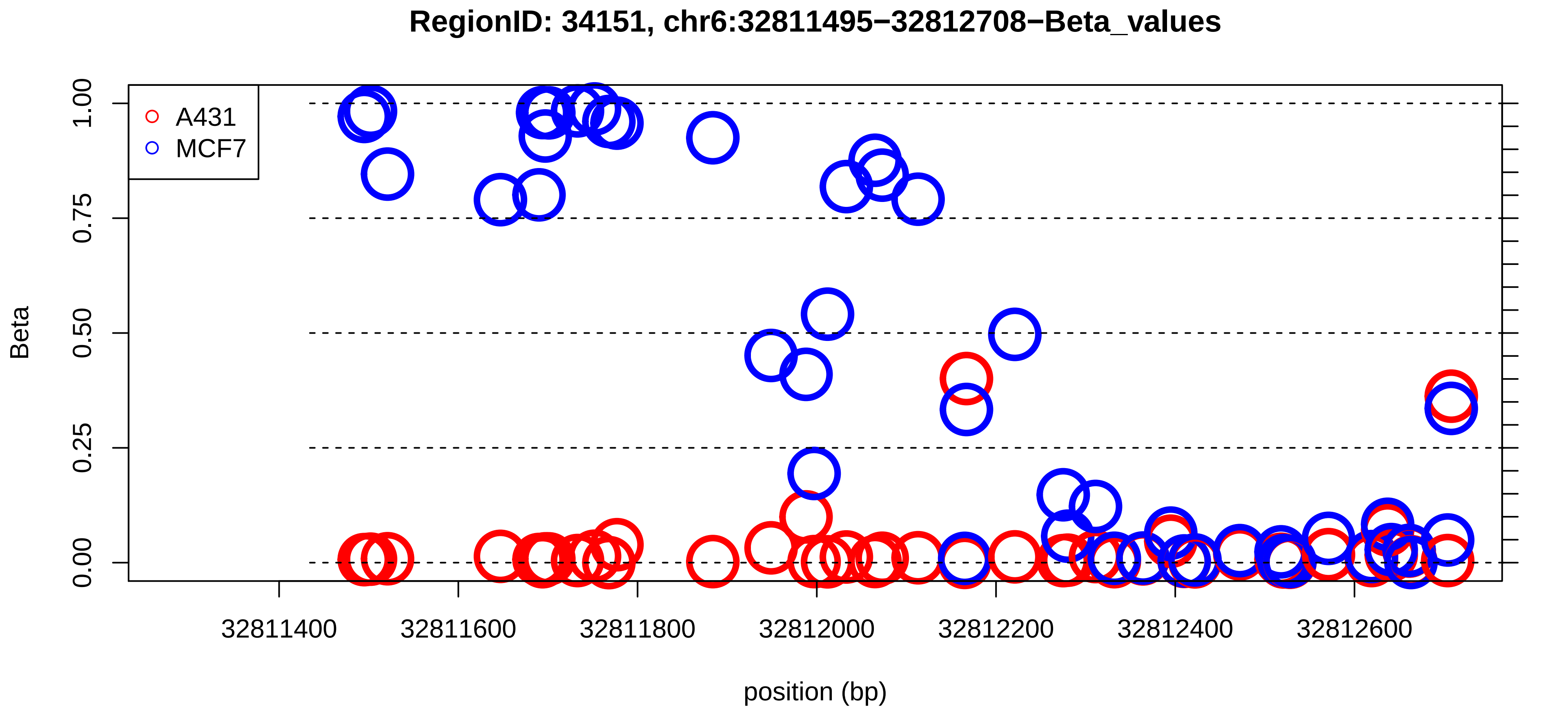

RegionID: 34151, chr6:32811495−32812708−Beta_values
A431
MCF7
Beta
1.00
0.75
0.50
0.25
0.00
32811400
32811600
32811800
32812000
32812200
32812400
32812600
position (bp)

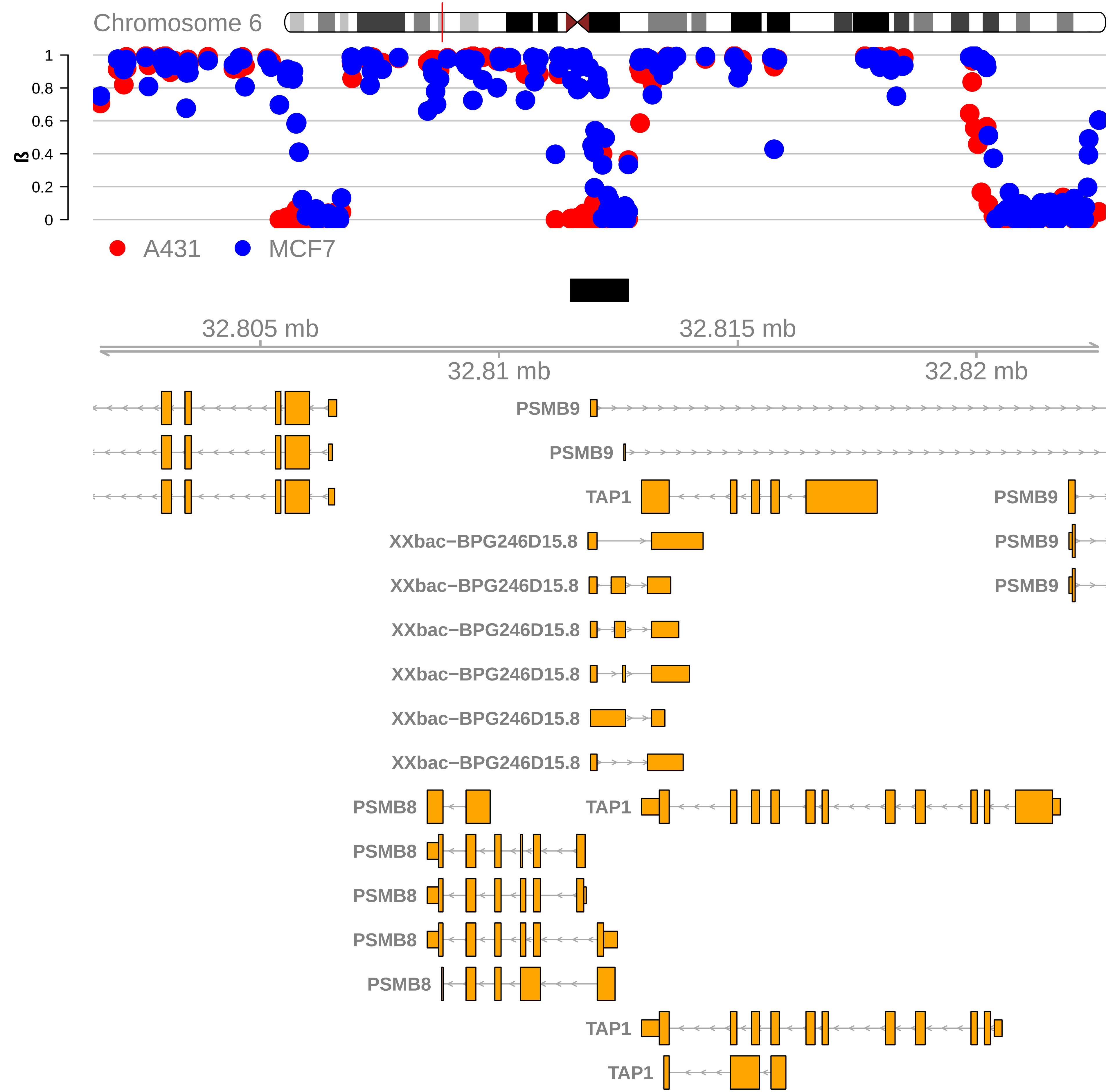

Chromosome 6
ß
1
0.8
0.6
0.4
0.2
0
A431
MCF7
32.805 mb
32.815 mb
32.81 mb
32.82 mb
PSMB9
PSMB9
TAP1
PSMB9
XXbac−BPG246D15.8
PSMB9
XXbac−BPG246D15.8
PSMB9
XXbac−BPG246D15.8
XXbac−BPG246D15.8
XXbac−BPG246D15.8
XXbac−BPG246D15.8
PSMB8
TAP1
PSMB8
PSMB8
PSMB8
PSMB8
TAP1
TAP1
